# Draft Genome Sequence of the Planktic Cyanobacterium *Tychonema bourrellyi*, Isolated from Alpine Lentic Freshwater

**DOI:** 10.1128/genomeA.01294-17

**Published:** 2017-11-22

**Authors:** Federica Pinto, Adrian Tett, Federica Armanini, Francesco Asnicar, Adriano Boscaini, Edoardo Pasolli, Moreno Zolfo, Claudio Donati, Nico Salmaso, Nicola Segata

**Affiliations:** a Centre for Integrative Biology, University of Trento, Trento, Italy; b Edmund Mach Foundation, San Michele all’Adige, Italy

## Abstract

We describe here the draft genome sequence of the cyanobacterium *Tychonema bourrellyi*, assembled from a metagenome of a nonaxenic culture. The strain (FEM_GT703) was isolated from a freshwater sample taken from Lake Garda, Italy. The draft genome sequence represents the first assembled *T. bourrellyi* strain.

## GENOME ANNOUNCEMENT

*Tychonema bourrellyi* (J. W. G. Lund) (1) is a filamentous cyanobacterium historically found in northern regions, such as northern Europe and Canada (2). *Tychonema* is a cold-stenotherm genus with thin filaments (4 to 6 µm wide) and mainly colonizes pelagic freshwater environments. *T. bourrellyi* is an important cyanobacterial species, since some strains can produce the neurotoxins anatoxin-a (ATX) and homoanatoxin-a (HTX), which can be harmful for human and animal health. Recently, Salmaso et al. (3) found that *T. bourrellyi* is spreading and diversifying in Alpine freshwater lakes, with 49 distinct strains being identified in lakes previously dominated by *Planktothrix* species. However, despite the relevance of this species for human health, no sequenced *T. bourrellyi* strains are currently available.

We reconstructed the draft genome of *T. bourrellyi* from a nonaxenic culture. The culture originated from a water sample collected in summer 2014 by vertical tows from 20 to 30 m to the surface with a plankton net at Lake Garda. A single filament of *Tychonema* was isolated under the microscope and, after being washed repeatedly with Z8 Cellstar medium (Greiner Bio-One GmbH, Germany), was incubated at 20°C at constant-light illumination (3). The *Tychonema* culture was filtered through 0.22-µm filters, and the PowerWater DNA isolation kit (Qiagen) was then used to extract the DNA. The paired-end libraries (Illumina) were prepared and run on an Illumina HiSeq 2500 platform (100-nucleotide [nt]-long reads). Raw reads were assembled using metaSPAdes version 3.10.1 (4), with default parameters, which generated 7,029 contigs larger than 1,000 bp, with a total size of 74 Mbp. From this assembly, the draft genome of *T. bourrellyi* was binned using the manually supervised anvi’o protocol based on the abundance and tetranucleotide frequency distributions (5), quality controlled by CheckM (6) (95% predicted completeness with no indication of contamination or strain heterogeneity), and scaffolded through SSPACE (7).

The assembled draft genome was confirmed to belong to the *T. bourrellyi* species by PhyloPhlAn analysis (8) and BLASTn searches of the 16S and *rbcLX* genes, which were 98.7% and 100% identical, respectively, to previously reported *T. bourrellyi* genes (3). The genome is 5.08 Mb assembled into 271 scaffolds, with an *N*_50_ value of 29,997 bp and a GC content of 44.63%. The genome annotation was performed with Prokka version 1.11 (9), which identified 4,493 coding sequences and 49 tRNAs. A candidate of a polyketide synthase family protein gene cluster potentially linked with the neurotoxic activity was identified in the genome at 40% amino acid identity (over an alignment length of 50%).

To our knowledge, this work represents the first sequencing of a *Tychonema bourrellyi* genome. Further analysis of the anatoxin genes involving more strains of this species are needed to unravel the genetic basis of *T. bourrellyi* toxicity.

### Accession number(s).

This genome project has been deposited at DDBJ/ENA/GenBank under the accession number NXIB00000000. The version described in this paper is version NXIB02000000.
